# Discordant gene expression in subcutaneous adipose and skeletal muscle tissues in response to exercise training

**DOI:** 10.14814/phy2.15995

**Published:** 2024-04-01

**Authors:** Michael Svensson, Alen Lovric, Torbjörn Åkerfeldt, David Hellsten, Tara Haas, Thomas Gustafsson, Eric Rullman

**Affiliations:** ^1^ Department of Community Medicine and Rehabilitation, Section of Sports Medicine Umeå University Umeå Sweden; ^2^ Department of Laboratory Medicine, Division of Clinical Physiology Karolinska Institutet, and Unit of Clinical Physiology, Karolinska University Hospital Stockholm Sweden; ^3^ Department of Medical Sciences, Clinical Chemistry Uppsala University Uppsala Sweden; ^4^ Department of Surgical and Perioperative Sciences Umeå University Umeå Sweden; ^5^ Faculty of Health, School of Kinesiology and Health Science York University Toronto Canada

**Keywords:** adipose tissue, GTEx, mRNA‐sequencing, obesity, skeletal muscle

## Abstract

Exercise has different effects on different tissues in the body, the sum of which may determine the response to exercise and the health benefits. In the present study, we aimed to investigate whether physical training regulates transcriptional network communites common to both skeletal muscle (SM) and subcutaneous adipose tissue (SAT). Eight such shared transcriptional communities were found in both tissues. Eighteen young overweight adults voluntarily participated in 7 weeks of combined strength and endurance training (five training sessions per week). Biopsies were taken from SM and SAT before and after training. Five of the network communities were regulated by training in SM but showed no change in SAT. One community involved in insulin‐ AMPK signaling and glucose utilization was upregulated in SM but downregulated in SAT. This diverging exercise regulation was confirmed in two independent studies and was also associated with BMI and diabetes in an independent cohort. Thus, the current finding is consistent with the differential responses of different tissues and suggests that body composition may influence the observed individual whole‐body metabolic response to exercise training and help explain the observed attenuated whole‐body insulin sensitivity after exercise training, even if it has significant effects on the exercising muscle.

## 1 INTRODUCTION

Exercise exerts different effects on different tissues in the body, which in sum determine the exercise response. There may be several reasons for the cell or tissue specific responses, such as that some cells are actively contracting during an activity while others do not, but also that the same stimulus may cause different responses in different cells. Both adipose tissue and skeletal muscle (SM) play important roles in metabolic health, and changes in both tissues are thought to be key to understanding the health benefits of exercise (Hawley et al., 2014). Adaptations following training are well documented in SM and include broad alterations to cellular signaling pathways and the SM transcriptome, most of which lead to improvements in contractile and metabolic functions (Hawley et al., 2014; Phillips et al., 2013). The effects of exercise on adipose tissue are far less well described than those on SM, but reductions in the size of individual adipocytes have been reported (Stinkens et al., 1985). Although subcutaneous adipose tissue (SAT) is not directly activated by exercise to the same extent as SM, transcriptome studies have reported altered SAT gene expression in response to exercise (Lam et al., 2016). Energy deprivation has been proposed as a key stimulus for SAT adaptation. However, recent research challenges this idea, showing that the adipose tissue transcriptome responds differently to caloric restriction than to exercise (Lam et al., 2016). Another plausible mechanism by which adipose tissue may be affected by exercise is cellular cross‐talk both within adipose tissue and via molecules from other tissues. Numerous circulating hormones (Hackney & Lane, 2015), metabolites, or myokines (Severinsen & Pedersen, 2020) released from SM are plausible candidates. Overall, systemic increases in these factors from exercise may differentially affect gene expression in different cell types, including noncontracting cells.

In the current study, we; (1) defined co‐expression network shared by SAT and SM, (2) explored how the gene communities comprising this shared network are regulated by exercise in the two tissues, and (3) determined if exercise regulated network communities in either SM or SAT relate to any clinical traits of T2D. All together, we hypothesized that by identifying common exercise regulated network communities with clinically meaningful biological functions in SAT and the SM, importance of exercise as a health stimulus could be better characterized in these two tissues.

## 2 STUDY DESIGN AND SUBJECTS

This Umea cohort study consists of a prospective cohort comprising sedentary (no regular exercise for at least 12 months before the study) men (*n* = 9) and women (*n* = 9) with overweight and obesity but without diagnosed metabolic disease, who underwent controlled exercise training for 7 weeks. The experimental study protocol was conducted in accordance with the Declaration of Helsinki and approved by the Regional Ethical Review Board, Umeå, Sweden (Dnr.13/262–31). Body composition (total body mass, total fat mass, and fat‐free mass) was determined using intelligent dual‐energy x‐ray absorptiometry technology (iDXA, GE Healthcare, Madison, WI, USA). Table 1 shows the characteristics of the subjects (baseline age 26.3 ± 3.0 years and BMI 30.3 ± 2.4, mean ± SD) before and after completion of the intervention. The training protocol included 7 weeks of combined strength and conditioning training, 1 h of training four to five times per week, of which three training sessions were supervised by an experienced personal trainer. The other two training sessions per week were performed outdoors and involved power walking with poles, 1 h each at a heart rate of 120–140 bpm. Training intensity was individualized and determined based on initial test results and progress during the training period. Participants were required to participate in at least 90% of the training sessions during the intervention period to be included in the per‐protocol analysis. This resulted in an increase in aerobic capacity and a significant increase in muscle strength. Total body mass remained unchanged, but with an increase in lean body mass and a decrease in fat mass. No significant change was observed in blood glucose, insulin, C‐peptide, and HOMA‐IR in the fasting state (Table 1).

**TABLE 1:** Subject characteristics. Data are presented as median, first and third quartile. Significant difference between pre and post indicated (Wilcoxon Signed Rank test).

	Pre	Post	*p*‐value
Total body mass (kg)	92.3 (84.3, 100.7)	91.5 (83.4, 99.7)	NS
Total fat mass (kg)	35.7 (31.2, 42.0)	34.2 (29.1, 42.1)	<0.001
Total lean mass (kg)	53.0 (45.4, 57.9)	53.4 (46.5, 59.8)	<0.001
VO_2max_ (L/min)	3.3 (2.8, 3.7)	3.7 (3.0, 4.0)	<0.001
Leg press (kg)	325 (170.0, 347.5)	450 (330.0, 663.3)	<0.001
Blood glucose (mmol/L)	5.2 (5.1, 5.5)	5.5 (5.0, 5.8)	NS
Insulin (mU/L)	13.3 (8.9, 19.9)	9.7 (7.3, 15.1)	NS
C‐peptide (nmol/L)	0.7 (0.6, 1.0)	0.6 (0.5, 0.9)	NS
HOMA‐IR	3.3 (2.1, 4.7)	2.3 (1.9, 3.4)	NS

Biopsies were taken from both SM and SAT simultaneously after an overnight fast and 48 h after the last exercise session. RNA was isolated from both tissues using Trizol (Thermo Fisher) and subsequently cleaned using RNAEasy columns (Qiagen). RNA concentration and integrity (RIN >7) were analyzed using Bioanalyzer (Agilent). Trueseq Poly‐A enriched libraries (Illumina) were prepared from 200 ng of total RNA and libraries were pair‐end sequenced using Nextseq 150 cycles in a total of four flow cells (Illumina). RNA extraction failed in samples from two subjects due to small amounts of tissue, so complete samples were collected from SM and SAT in 16 subjects. Raw sequencing data were submitted to FASTQCR (v0.1.2) for quality control and aligned to the whole human transcriptome (GRCh38) using the Kallisto sequence pseudo‐aligner (v0.44.0) using the default options. After trimmed mean M‐values normalization, expression matrices were converted to log2CPM values using edgeR (v3.24.3) and filtered for genes with low average expression (log2CPM <4). All RNA sequencing data is available through the Gene Expression Omnibus https://www.ncbi.nlm.nih.gov/geo/.

## 3 BUILDING MULTI‐TISSUE INTEGRATED CO‐EXPRESSION NETWORK

To enable an integrated transcriptome analysis of both SAT and SM, a co‐expression network of genes expressed in both tissues was constructed (Figure S1) as follows: RNA‐seq count data from human SAT (*n* = 442) and SM (*n* = 564) tissues was obtained from GTEx database (GTEx; https://gtexportal.org/home/) version 7 and pre‐processed in the same way as described above (Law et al., 2016). Candidate genes were selected based on confirmed SM and SAT mRNA expression in both GTEx and the Umea dataset (average log2CPM >4) and only genes with confirmed expression at the protein level in either tissue, based on the Human Protein Atlas (Digre & Lindskog, 2020), were considered. For each tissue, gene pairs were then selected based on the Pearson correlation coefficient (*R* > 0.30 and FDR <1%) and used to construct the network with the co‐expressed genes representing both tissues. The result was a final network with 1169 genes, 5267 connections, and an overall high correlation strength, *R*_mean_ = 0.64. The network was subdivided into smaller subsets of highly connected genes (communities), utilizing Louvain algorithm (Blondel et al., 2008) relying on network connectivity. Functional enrichment analysis for biological processes (BP) and KEGG pathway was conducted for the whole network as well as for each community by using web‐based Database for Annotation, Visualization, and Integrated Discovery (DAVID) v6.8 (https://david.ncifcrf.gov/). Ingenuity pathway analysis (IPA) was used to identify putative key up‐stream regulators of the identified communities. IPA v7.6 was used and a *q*‐value <0.05 was considered as significant. A descriptive title for each community was selected from one of the enriched GO, KEGG, or upstream regulator terms. Eight communities were identified; 233 genes associated with processes related to “Extracellular matrix organization” (GO:0030198; FDR <0.001), with TGFB being the major upstream regulator, 147 genes related to “Glucose‐insulin‐AMPK”, with an upstream regulator of AMPK and insulin signaling (hsa04910; FDR <0.028), 178 genes associated with “Fatty acid metabolism” (GO:0006635; FDR <0.001), 41 genes associated with “Angiogenesis” (GO:0001525; FDR <0.001) and “Integrin signaling”, 106 genes was enriched in “Calcium signaling” and “Muscle contraction” (GO:0006936; FDR <0.001) and 32 genes was related to “Actin cytoskeletal signaling” and “Muscle filament sliding” (GO:0030198; FDR <0.001), with MEF2C as a key upstream regulator. The smallest community of 19 genes were enriched for “Transcriptional regulation” processes (GO:0045944; FDR <0.001), with MAPK as an upstream regulator. The complete list of genes and communities are given in Tables S1 and S2.

## 4 ANALYSIS OF NETWORK COMMUNITES IN RELATION TO EXERCISE

**FIGURE 1:**
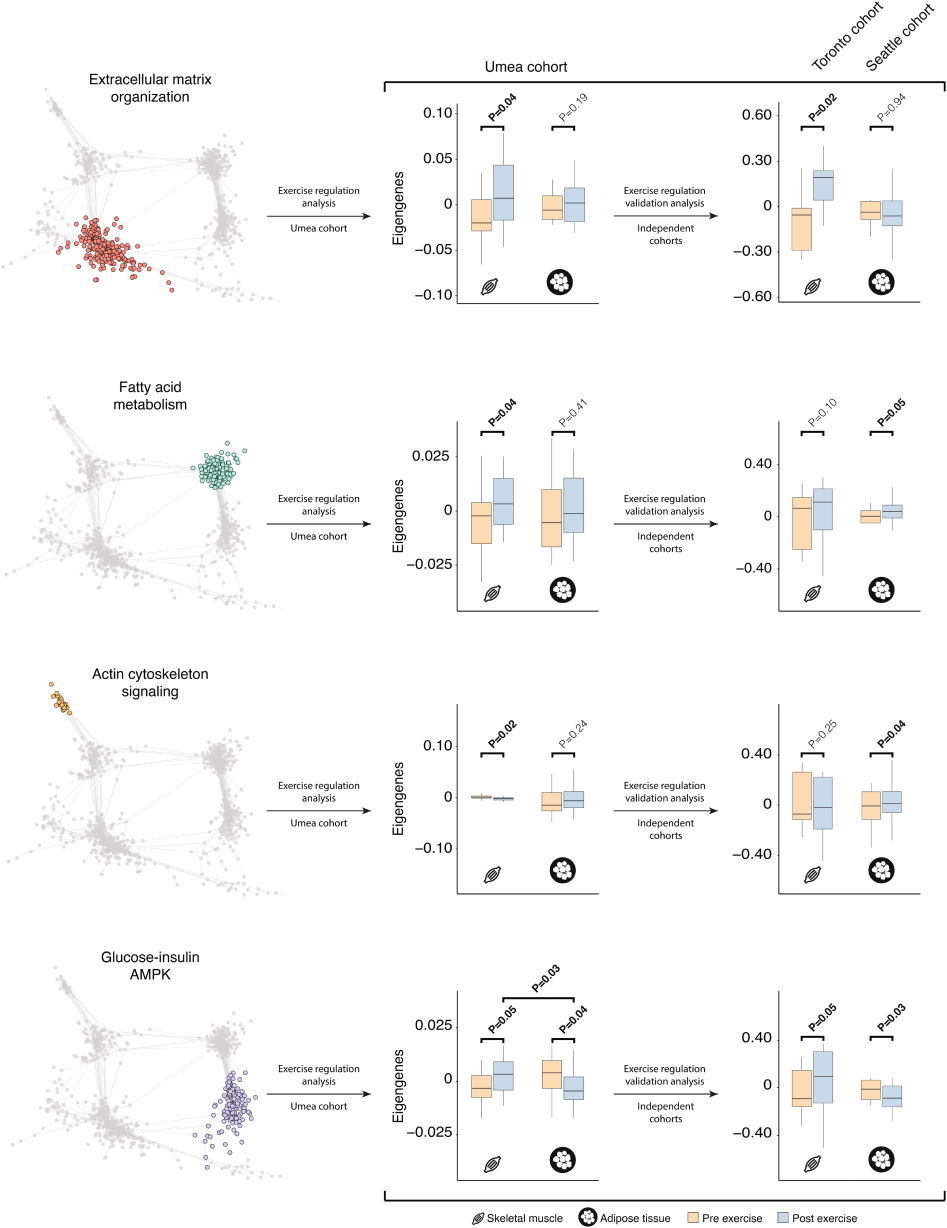
Transcritpional effects of 7 weeks of high‐intensity exercise and association with phenotypical traits. The effect of the exercise intervention on transcriptional regulation of network communities in Umea cohort and two independent studies (Toronto cohort—muscle tissue; Seattle cohort—adipose tissue).

To study tissue‐specific transcriptional regulation by exercise the network communities were analyzed pre exercise and 48 h after the last exercise bout in the Umea cohort. The study‐design with paired, complementary SM and SAT biopsies in combination with identification of gene communities with similar patterns of expression at baseline, allowed for an integrated cross‐tissue analysis of the exercise effects by using two‐way repeated measures ANOVA (R version 4.1.0) in the form of Time × Tissue. A *p*‐value of 0.05 for the interaction term was considered significant. There was a higher number of communities regulated by exercise in SM (*n* = 5) than in SAT (*n* = 1), (Table 2). The communities enriched for “Fatty acid metabolism”, “Angiogenesis”, “Extracellular matrix organization”, and “Calcium signaling” showed significant regulation only in SM. The community enriched for “Glucose‐insulin‐AMPK” presenting significant changes in both tissues, up‐regulated in SM (FC = 0.62, *p* = 0.048) and down regulated in SAT (FC = −0.45, *p* = 0.039) with a *p*‐value for Time × Tissue of 0.031. The opposing regulation was also reflected at the level of individual genes for key members of this community: For example, fatty acid synthase (FASN) was highly expressed in adipose tissue before exercise but was downregulated in post‐exercise samples, whereas FASN expression was low in SM before exercise and increased after exercise (*p*‐value for Tissue × Time 0.04), (Figure S2; Montastier et al., 2015). To validate the results, a publicly available dataset was used: For SM, we used the Toronto cohort, a recent RNA sequencing dataset (GSE163356) of SM transcriptome before and after a 3‐week sprint training (nine HIIT sessions consisting of 10 × 4 min cycling at ~83% of VO_2peak_ intensity) in healthy young men (*n* = 11, 24.8 ± 1 year, height of 180.4 ± 1.8 cm, 75.5 ± 3.4 kg weight, and body mass index of 23.2 ± 0.8 kg/m2) (Norrbom et al., 2022). The majority of network genes identified in the Umea cohort could be mapped to this data set (1051/1069, 98%). Using paired *t*‐tests, four network communities, Extracellular matrix organization’, “Glucose‐insulin‐AMPK”, “Angiogenesis”, “Calcium signaling”, showed analogous regulation in the two datasets (Figure 1, Table 2). The “Fatty acid metabolism” and “Actin cytoskeleton signaling” community, which were regulated by exercise in the Umea study, could not be confirmed in the Toronto study but showed similar numerical trends. The publicly available Seattle study consists of microarray data from SAT obtained from 14 overweight middle‐aged women before and after a 6‐month exercise intervention (45 min of moderate to intense exercise at least 5 days per week) (Campbell et al., 2013). The majority of transcripts in our co‐expression network (80% of the 1069 transcripts) could be mapped to the Seattle data set (Table S2). After normalization and eigengene aggregation, each community was analyzed for differential expression before and after the training intervention. It was confirmed that the “Glucose‐insulin‐AMPK” in the Seattle cohort was negatively regulated by training. Two communities “Actin cytoskeletal signaling” and “Cellular senescence” were upregulated and downregulated, respectively, but were not significantly altered in the Umea cohort (Figure 1, Table 2).

**TABLE 2:** Transcriptional regulation after 7 weeks of intense exercise training. In the Umea cohort and Seattle (SAT) and Toronto (SM) cohorts.

Community	Umea cohort					Toronto cohort		Seattle cohort	
	FC	*p*‐value muscle	FC	*p*‐value adipose	*p*‐value tissue interaction	FC	*p*‐value muscle	FC	*p*‐value adipose
Extracellular matrix organization	UP	0.04*	NS	0.19	0.11	UP	0.02*	NS	0.94
Fatty acid metabolism	UP	0.04*	NS	0.41	0.51	NS	0.10	UP	0.05*
Glucose‐insulin‐AMPK	UP	0.05*	DOWN	0.04*	0.03*	UP	0.05*	DOWN	0.03*
Calcium signaling	DOWN	0.04*	NS	0.08	0.03*	DOWN	0.01*	NS	0.96
Cellular senescence	NS	0.71	NS	0.77	0.95	NS	0.06	DOWN	0.02*
Angiogenesis, integrin signaling	UP	0.05**	NS	0.99	0.1	UP	0.03*	NS	0.82
Actin cytoskeleton signaling	DOWN	0.02*	NS	0.24	0.11	NS	0.25	UP	0.04*
Transcriptional regulation	NS	0.92	NS	0.43	0.41	NS	0.76	NS	0.44

* Indicates *p* < 0.05.

** Indicates borderline significance with *p* = 0.053.

## 5 RELATIONSHIP BETWEEN NETWORK COMMUNITIES AND CLINICAL TRAITS

**FIGURE 2:**
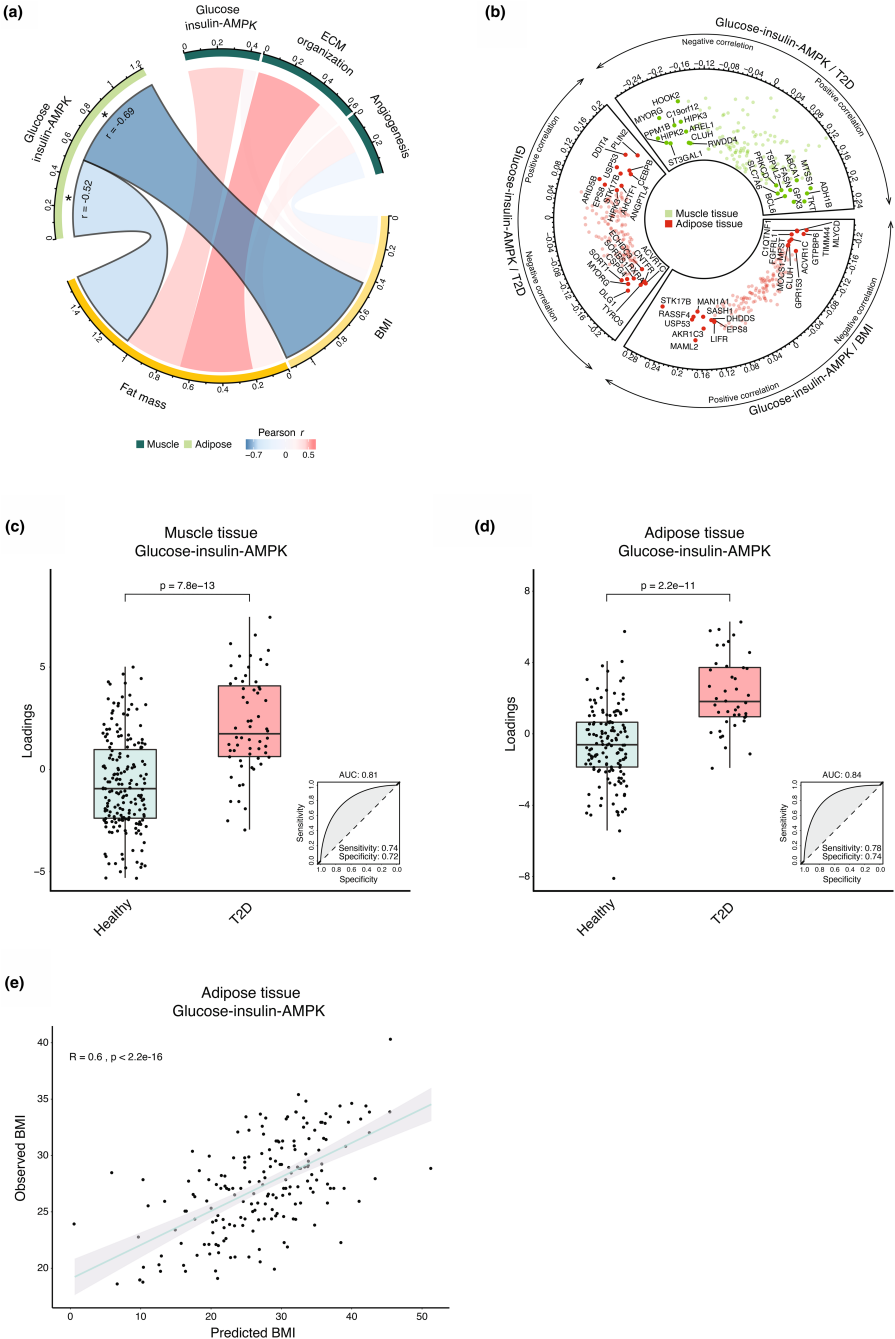
Association between relevant phenotypical traits and gene expression of the network. (a) Association with phenotypical traits in the Umea cohort of exercise induced network communites (b) “Glucose‐insulin‐AMPK”, expression modeled against BMI and T2D prevalence in GTEx cohort using OPLS regression. (c, d) OPLS‐model accuracy for class separation of patients with T2D from healthy individuals in GTEx cohort considering gene expression of “Glucose‐insulin‐AMPK” in SAT and SM (e) based on GTEx cohort SAT expression of the genes in the “Glucose‐insulin‐AMPK” community in relation to BMI.

Gene expression of each community was decomposed using the first principal component of expression levels that is, eigengenes (Langfelder & Horvath, 2007) and the associations in the Umea cohort with clinical traits BMI, fat mass, C‐peptide, glucose, insulin and HOMA were tested using Pearson regression between eigengenes and clinical traits of interest. The “Glucose‐insulin‐AMPK” community was negatively associated with BMI (*R* = −0.69, *p* < 0.01) and total fat mass (*R* = −0.52, *p* = 0.04), (Figure 2a). Conversely, SM gene expression in the Umea cohort was not significantly correlated with any of the tested clinical characteristic. To further assess the clinical relevance of the “Glucose‐insulin‐AMPK” expression, we used GTEx dataset (http://www.ncbi.nlm.nih.gov/gap; dbGaP accession number phs000424.v7.p2.). While the size of the GTEx cohort is substantial, the clinical characterization of individual subjects is limited. Therefore, the analysis was limited to BMI and prevalence of T2D, controlling for sex and age. All available observations were used, except intensive‐care respirator cases (GTEx HARDY‐scale of 0), in a total of 188 and 252 subjects for SAT and SM, respectively. “Glucose‐insulin‐AMPK” expression was modeled against BMI by means of OPLS regression using the OPLS‐library in R (Thevenot et al., 2015) and prevalence of T2D by OPLS‐DA (as it is a dichotomous variable) The contribution of each individual gene in relation to the outcomes was obtained as Variable Importance in Projection (VIP). To minimize overfitting, the model was cross‐validated 1000 times and a resulting *q*‐value <0.05 for the model was considered significant. Performance of network communities in classification of T2D was estimated by using Area Under the Receiver Operating Characteristics Curves (AUROC). In the SAT, an association between the “Glucose‐insulin‐AMPK” community and BMI was identified, where the final model had a *R* ^2^ of 0.37 and a cross‐validated *p*‐value of <0.01. Fifty‐one genes in the community were strong predictors (VIP >1) among which STK17B, USP53, and MAN1A1 contributed heavily to this relationship, see Figure 2b,e. SAT expression of the “Glucose‐insulin‐AMPK” community showed significant predictive value against prevalence of T2D, with higher expression among individuals diagnosed with T2D (*p* < 0.001) in both SM muscle and SAT (Figure 2c,d). The classification model had *R* ^2^ of 0.25 with a cross‐validated *p*‐value of <0.01, identifying USP53, PLIN2, and CEBPB as genes with high contribution to the class difference, see Figure 2b. In SM, the OPLS‐regression found no significant correlation between the “Glucose‐insulin‐AMPK” community and BMI (*R* ^2^ = 0.18 and *p* > 0.05) but there was a clear separation with regards to T2D with *R* ^2^ of 0.23 and *p*‐value of <0.01 after cross‐validation. Several genes previously reported in relation to T2D had high variable importance including FASN, TKT and ADH1B, see Figure 2b. The VIP and *R*‐value for all genes are reported in Table S3.

## 6 DISCUSSION

It is well known that various tissues adapt uniquely to physical activity, ultimately resulting in distinct effects on individual performance and potential health outcomes. The current study aimed to develop a strategy to analyze exercise‐induced effects on distinct and clinically significant biological functions in two key tissues in a clinically relevant population. To facilitate this, coexpression network analysis, a known powerful strategy to cluster genes into biologically relevant communities was used to identify eight communities of genes involved in BP and pathways known to play important roles in both SM and SAT. Training had a more pronounced direct effect on gene expression in SM at the gene network community level, consistent with known effects of training on SM and the results were also validated in the Toronto cohort. This suggests that the internal stimuli or responses elicited by training are specific to different cell types in defined, co‐expressed BP. Biopsies of the two tissues in question were taken from the same anatomical location in the Umea cohort, so the systemic external effects of exercise on the different tissues should be similar.

An important observation in the current study was the inverse regulation of the “Glucose‐insulin‐AMPK” community in response to exercise in SAT compared with SM, and confirmed in the Seattle cohort. Members of this community include genes with established associations with insulin signaling, such as AKT3, IRS2, FOXO1, and FASN. Robust upregulation of glucose and fat catalytic machinery and glucose metabolism‐related pathways in SM was counterbalanced by significant downregulation in SAT. The tissue‐specific aberrant regulation observed in our study at the level of the network community was also reflected at the level of individual genes for key members of the community such as FASN, a cytosolic enzyme critical for the de novo synthesis of long‐chain fatty acids. In support of our findings, FASN gene expression has been reported to increase in human SM after both high‐intensity training and 6 weeks of endurance training, with intra‐myocellular lipids (IMCL) accumulating in muscle fibers (Summermatter et al., 2010) coherent with the numerous studies that show IMCL levels in humans increase with training (Goodpaster et al., 2001). Consequently, the tissue interaction effect suggesting a decrease in FASN expression in adipose tissue could lead to decreased lipogenesis from glucose and thus decreased adipocyte mass.

By demonstrating that the effects of exercise training differ in different tissues, our results extend previous reports describing differential effects of acute exercise training on insulin sensitivity and glucose utilization when comparing trained and untrained muscles and supports the findings by Steenberg et al (Steenberg et al., 2020). Our results also confirm other reports, for example that physical training improves insulin‐dependent glucose uptake in SM but not in adipose tissue in healthy overweight subjects (Reichkendler et al., 2013). The clinical relevance of our observation was also demonstrated by the significant association between “Glucose‐insulin‐AMPK” expression with both BMI and presence of diabetes in the GTEx cohort, and also correlated significantly with BMI and total fat mass in the Umea cohort. This provides an important perspective of relevant cross‐tissue/integrated metabolic effects of exercise interventions. For example, it could explain the results of several studies that have shown no improvement in whole‐body insulin sensitivity after exercise training, although there were significant effects on the exercising muscle (Reichkendler et al., 2013; Steenberg et al., 2020).

This study has a few potential limitations: First, the original study‐cohort in which the exercise intervention was conducted has a small sample size and the average effect‐size with regards to exercise effects was small wherefore we might underestimate the transcriptional effects of exercise training herein. The cohort utilized for validation of the transcriptional response in adipose tissue was also quite different both in terms of age, sex, type and duration of exercise. This is due to the scarcitiy of publicly available adipose tissue transcriptional data in relation to exercise. It is whoever worth noting that despite these differences of the Umea and Seattle cohorts the transcpriptional regulation of the “Glucose‐insulin‐AMPK signaling” community was similar indicating that this is a conserved and general adipose tissue response to exercise training.

In conclusion, physical exercise leads to different, opposite effects on the transcripts involved in “Glucose‐insulin‐AMPK” signaling in muscle and adipose tissue of obese individuals. This may also explain the observation that increased whole‐body insulin sensitivity is sometimes absent after exercise, although increased SM insulin sensitivity has been documented simultaneously. It also suggests that individual body composition may influence the metabolic effects of exercise.

## FUNDING INFORMATION

Eric Rullman was partly supported by the Swedish Heart and Lung foundation (20220725).

## ETHICS STATEMENT

The subjects were informed about the experimental procedures and the nature of the training program before consenting to participate. The study was performed in accordance with the Declaration of Helsinki and the national Swedish Ethical Review Authority approved the protocol and experimental procedures.

## Supporting information


Figure S1:

Figure S2:

Table S1:

Table S2:

Table S3:

## References

[phy215995-bib-0001] Blondel, V. D. , Guillaume, J. L. , Lambiotte, R. , & Lefebvre, E. (2008). Fast unfolding of communities in large networks. Journal of Statistical Mechanics: Theory and Experiment, 2008, P10008.

[phy215995-bib-0002] Campbell, K. L. , Foster‐Schubert, K. E. , Makar, K. W. , Kratz, M. , Hagman, D. , Schur, E. A. , Habermann, N. , Horton, M. , Abbenhardt, C. , Kuan, L. Y. , Xiao, L. , Davison, J. , Morgan, M. , Wang, C. Y. , Duggan, C. , McTiernan, A. , & Ulrich, C. M. (2013). Gene expression changes in adipose tissue with diet‐ and/or exercise‐induced weight loss. Cancer Prevention Research (Philadelphia, Pa.), 6, 217–231. 23341572 10.1158/1940-6207.CAPR-12-0212 PMC3738189

[phy215995-bib-0003] Digre, A. , & Lindskog, C. (2020). The Human Protein Atlas–spatial localization of the human proteome in health and disease. Protein Science, 30(1), 218–233. 33146890 10.1002/pro.3987 PMC7737765

[phy215995-bib-0004] Goodpaster, B. H. , He, J. , Watkins, S. , & Kelley, D. E. (2001). Skeletal muscle lipid content and insulin resistance: Evidence for a paradox in endurance‐trained athletes. The Journal of Clinical Endocrinology and Metabolism, 86, 5755–5761. 11739435 10.1210/jcem.86.12.8075

[phy215995-bib-0005] Hackney, A. C. , & Lane, A. R. (2015). Exercise and the regulation of endocrine hormones. Progress in Molecular Biology and Translational Science, 135, 293–311. 26477919 10.1016/bs.pmbts.2015.07.001

[phy215995-bib-0006] Hawley, J. A. , Hargreaves, M. , Joyner, M. J. , & Zierath, J. R. (2014). Integrative biology of exercise. Cell, 159(4), 738–749. 25417152 10.1016/j.cell.2014.10.029

[phy215995-bib-0007] Lam, Y. Y. , Ghosh, S. , Civitarese, A. E. , & Ravussin, E. (2016). Six‐month calorie restriction in overweight individuals elicits transcriptomic response in subcutaneous adipose tissue that is distinct from effects of energy deficit. The Journals of Gerontology. Series A, Biological Sciences and Medical Sciences, 71, 1258–1265. 26486851 10.1093/gerona/glv194 PMC6279208

[phy215995-bib-0008] Langfelder, P. , & Horvath, S. (2007). Eigengene networks for studying the relationships between co‐expression modules. BMC Systems Biology, 1, 54. 18031580 10.1186/1752-0509-1-54 PMC2267703

[phy215995-bib-0009] Law, C. W. , Alhamdoosh, M. , Su, S. , Dong, X. , Tian, L. , Smyth, G. K. , & Ritchie, M. E. (2016). RNA‐seq analysis is easy as 1‐2‐3 with limma, Glimma and edgeR. F1000Res, 5, 5. 10.12688/f1000research.9005.1 PMC4937821 27441086

[phy215995-bib-0010] Montastier, E. , Villa‐Vialaneix, N. , Caspar‐Bauguil, S. , Hlavaty, P. , Tvrzicka, E. , Gonzalez, I. , Saris, W. H. , Langin, D. , Kunesova, M. , & Viguerie, N. (2015). System model network for adipose tissue signatures related to weight changes in response to calorie restriction and subsequent weight maintenance. PLoS Computational Biology, 11, e1004047. 25590576 10.1371/journal.pcbi.1004047 PMC4295881

[phy215995-bib-0011] Norrbom, J. M. , Ydfors, M. , Lovric, A. , Perry, C. G. R. , Rundqvist, H. , & Rullman, E. (2022). A HIF‐1 signature dominates the attenuation in the human skeletal muscle transcriptional response to high‐intensity interval training. Journal of Applied Physiology (1985), 132(6), 1448–1459. 10.1152/japplphysiol.00310.2021 35482326

[phy215995-bib-0012] Phillips, B. E. , Williams, J. P. , Gustafsson, T. , Bouchard, C. , Rankinen, T. , Knudsen, S. , Smith, K. , Timmons, J. A. , & Atherton, P. J. (2013). Molecular networks of human muscle adaptation to exercise and age. PLoS Genetics, 9(3), e1003389. 23555298 10.1371/journal.pgen.1003389 PMC3605101

[phy215995-bib-0013] Reichkendler, M. H. , Auerbach, P. , Rosenkilde, M. , Christensen, A. N. , Holm, S. , Petersen, M. B. , Lagerberg, A. , Larsson, H. B. , Rostrup, E. , Mosbech, T. H. , Sjodin, A. , Kjaer, A. , Ploug, T. , Hoejgaard, L. , & Stallknecht, B. (2013). Exercise training favors increased insulin‐stimulated glucose uptake in skeletal muscle in contrast to adipose tissue: A randomized study using FDG PET imaging. American Journal of Physiology. Endocrinology and Metabolism, 305, E496–E506. 23800880 10.1152/ajpendo.00128.2013

[phy215995-bib-0014] Severinsen, M. C. K. , & Pedersen, B. K. (2020). Muscle‐organ crosstalk: The emerging roles of myokines. Endocrine Reviews, 41, 594–609. 32393961 10.1210/endrev/bnaa016 PMC7288608

[phy215995-bib-0015] Steenberg, D. E. , Hingst, J. R. , Birk, J. B. , Thorup, A. , Kristensen, J. M. , Sjoberg, K. A. , Kiens, B. , Richter, E. A. , & Wojtaszewski, J. F. P. (2020). A single bout of one‐legged exercise to local exhaustion decreases insulin action in nonexercised muscle leading to decreased whole‐body insulin action. Diabetes, 69, 578–590. 31974138 10.2337/db19-1010

[phy215995-bib-0016] Stinkens, R. , Brouwers, B. , Jocken, J. W. , Blaak, E. E. , Teunissen‐Beekman, K. F. , Hesselink, M. K. , van Baak, M. A. , Schrauwen, P. , & Goossens, G. H. (1985). Exercise training‐induced effects on the abdominal subcutaneous adipose tissue phenotype in humans with obesity. Journal of Applied Physiology, 2018(125), 1585–1593. 10.1152/japplphysiol.00496.2018 30212302

[phy215995-bib-0017] Summermatter, S. , Baum, O. , Santos, G. , Hoppeler, H. , & Handschin, C. (2010). Peroxisome proliferator‐activated receptor γ coactivator 1α (PGC‐1α) promotes skeletal muscle lipid refueling in vivo by activating de novo lipogenesis and the pentose phosphate pathway*. The Journal of Biological Chemistry, 285, 32793–32800. 20716531 10.1074/jbc.M110.145995 PMC2963391

[phy215995-bib-0018] Thevenot, E. A. , Roux, A. , Xu, Y. , Ezan, E. , & Junot, C. (2015). Analysis of the human adult urinary metabolome variations with age, body mass index, and gender by implementing a comprehensive workflow for univariate and OPLS statistical analyses. Journal of Proteome Research, 14, 3322–3335. 26088811 10.1021/acs.jproteome.5b00354

