# Supplementary figures and images for: Discordant gene expression in subcutaneous adipose and skeletal muscle tissues in response to exercise training
Source: Physiol Rep. 2024 Apr 1;12(7):e15995. doi: 10.14814/phy2.15995 (PMC10984804; doi:10.14814/phy2.15995)

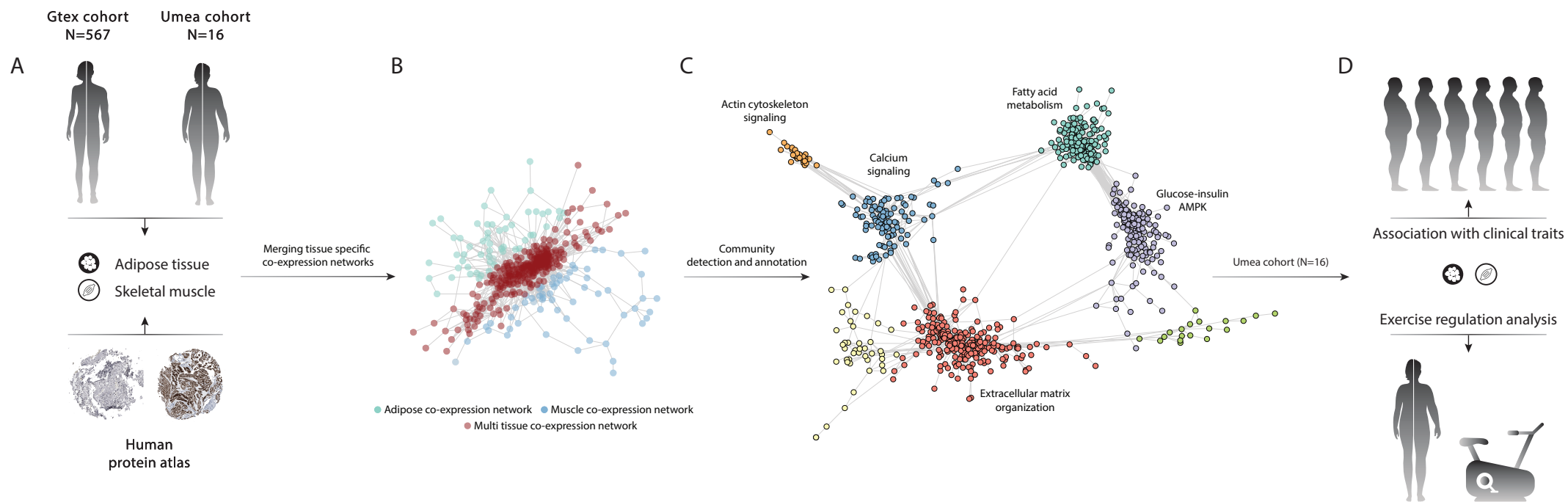

Supplement: Supplementary file 1 — Figure S1: [file PHY2-12-e15995-s001.pdf]

A

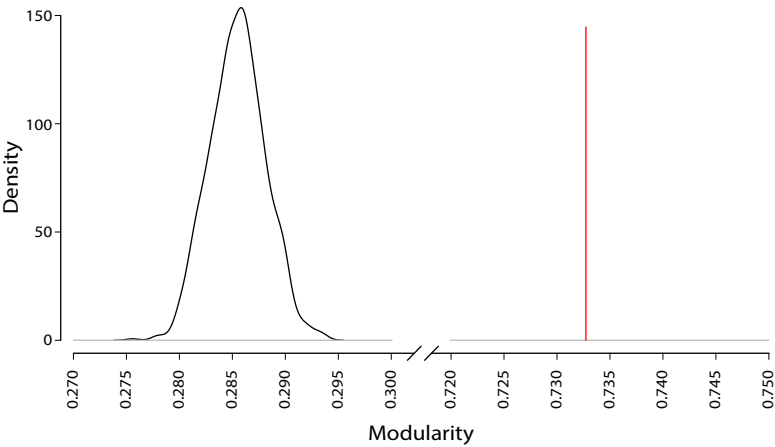

B

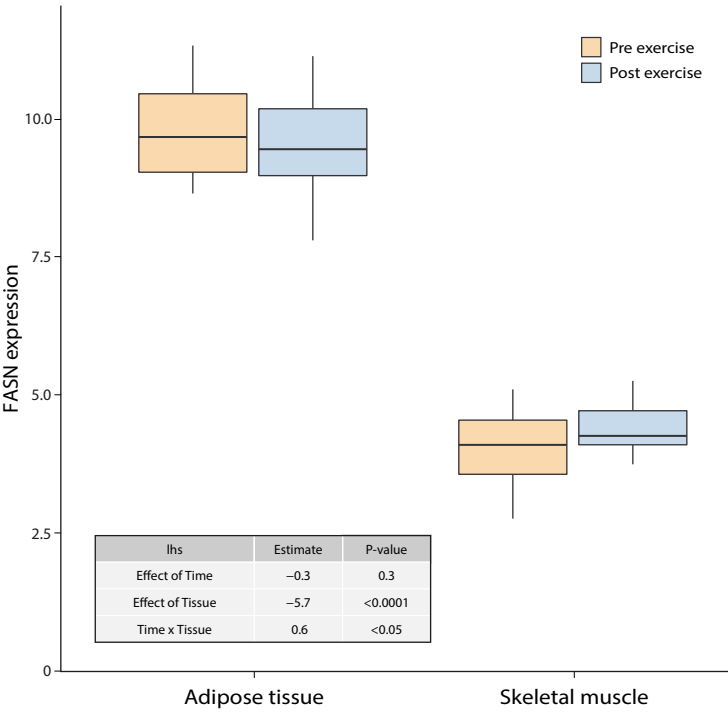

Supplement: Supplementary file 2 — Figure S2: [file PHY2-12-e15995-s002.pdf]
